# Effect of the food production chain from farm practices to vegetable processing on outbreak incidence

**DOI:** 10.1111/1751-7915.12178

**Published:** 2014-09-24

**Authors:** Yangjin Jung, Hyein Jang, Karl R Matthews

**Affiliations:** Department of Food Science, Rutgers, The State University of New JerseyNew Brunswick, NJ, 08901, USA

## Abstract

The popularity in the consumption of fresh and fresh-cut vegetables continues to increase globally. Fresh vegetables are an integral part of a healthy diet, providing vitamins, minerals, antioxidants and other health-promoting compounds. The diversity of fresh vegetables and packaging formats (spring mix in clamshell container, bagged heads of lettuce) support increased consumption. Unfortunately, vegetable production and processing practices are not sufficient to ensure complete microbial safety. This review highlights a few specific areas that require greater attention and research. Selected outbreaks are presented to emphasize the need for science-based ‘best practices’. Laboratory and field studies have focused on inactivation of pathogens associated with manure in liquid, slurry or solid forms. As production practices change, other forms and types of soil amendments are being used more prevalently. Information regarding the microbial safety of fish emulsion and pellet form of manure is limited. The topic of global climate change is controversial, but the potential effect on agriculture cannot be ignored. Changes in temperature, precipitation, humidity and wind can impact crops and the microorganisms that are associated with production environments. Climate change could potentially enhance the ability of pathogens to survive and persist in soil, water and crops, increasing human health risks. Limited research has focused on the prevalence and behaviour of viruses in pre and post-harvest environments and on vegetable commodities. Globally, viruses are a major cause of foodborne illnesses, but are seldom tested for in soil, soil amendments, manure and crops. Greater attention must also be given to the improvement in the microbial quality of seeds used in sprout production. Human pathogens associated with seeds can result in contamination of sprouts intended for human consumption, even when all appropriate ‘best practices’ are used by sprout growers.

## Introduction

Consumption of fresh vegetables has dramatically increased globally with the advent of new technologies. The wide selection of products from bagged salads to fresh-cut fruits now makes consumption of fresh fruits and vegetables by consumers affordable, easy and a tasty adventure. In 1998, a typical grocery store in the United States carried 345 produce items compared with just 173 in 1987 (Dimitri *et al*., 2003). Consumption of fresh produce is part of a healthy diet, but pathogen contamination of fresh produce has resulted in serious public health consequences. Outbreaks in recent years have been linked to tomatoes, spinach, lettuce and seed sprouts. There is no exclusion when it comes to fresh vegetables that can be contaminated by foodborne pathogens.

Globally, the number of outbreaks and cases of foodborne illness associated with the consumption of contaminated produce continues to escalate (Critzer and Doyle, 2010; Teplitski *et al*., 2011; Hoelzer *et al*., 2012). Fresh and fresh-cut vegetables are not processed in ways that will effectively eliminate human pathogens. The types of human pathogens that can be associated with produce are varied and may include bacteria (*Escherichia coli* O104:H4, *Listeria monocytogenes*, *Salmonella*), viruses (hepatitis A virus, norovirus) and parasites (*Cryptosporidium parvum*, *Cyclospora cayetanensis*). The sources of those pathogens and other microbes traverses the continuum from farm to plate and includes contaminated agricultural water, soil amendments, contaminated harvesting equipment, field workers, processing plants and retail handling.

The improper handling prior to and during processing can compromise the safety of a product. Although, the growth of pathogens including *L. monocytogenes*, *E. coli* O157:H7 and *Salmonella* will be limited or prevented by cooling crops to 4°C or less, their persistence in crop produce has been reported. The fate of *Salmonella* and *E. coli* O157:H7 on cilantro, basil, chive and other fresh herbs during refrigerated (4°C) storage was investigated (Hsu *et al*., 2006). Initial populations of both pathogens decreased rapidly, but the pathogens survived for at least 24 days of storage. Others have reported the ability of *E. coli* O157:H7 to grow on damaged spinach leaves held at 8°C, generally considered the minimum temperature for growth of that pathogen (Khalil and Frank, 2010). Developed and developing countries face challenges in developing and implementing measures that will improve the microbial safety of vegetables.

## Regulations

Establishment of new guidelines and regulations for vegetable processing and handling should bring about improvements in the microbial safety of fresh vegetables. Globally, considerable attention has been focused on the safety of fresh and fresh-cut fruits. In the United States, the Food Safety Modernization Act (FSMA) now requires the US Food and Drug Administration (USFDA) to establish science-based minimal standards for the safe production and harvesting of those types of raw fruits and vegetables (e.g. lettuce, tomatoes and cantaloupes) for which standards are necessary to minimize the risk to human health, including death. The ‘standards’ will focus on microbiological hazards because illnesses attributed to chemical and physical hazards associated with the consumption of fresh and fresh-cut produce are rare (Food and Drug Administration, 2013a,b).

Consumers in developed countries desire products year-round, regardless of the growing season of those commodities. In order to fulfil those demands, companies source products from throughout the world. The production and processing practices in developing countries may not achieve appropriate safety levels placing consumers within that country and throughout the world at risk of illness through export of those commodities. In the United States, under FSMA, foreign suppliers are required to meet the same standards as domestic producers. This also includes foreign farms that meet the criteria of ‘covered farms’ that grow, harvest, pack or hold covered produce for import into the United States. Meeting the standards outlined will likely be costly and may limit the ability of some developing countries to comply, impeding exports of fresh fruits and vegetables to the United States. Similar to the United States, the European Union and other developed countries have elaborate standards and guidelines to enhance the safety of food produced domestically and imported food (Jaud *et al*., 2013). Human health problems arise when best practices are not used throughout the farm-to-plate continuum, regardless of where the food is produced.

## Vegetable contamination along the food production chain

Vegetables can become contaminated with human pathogens at multiple points along the farm-to-table production/supply chain (Matthews, 2013). The microbial safety of produce is a concern; in the United States, between 1996 and 2010, approximately 23% of total outbreaks of foodborne illness were produce related. Bacterial agents (e.g. *Salmonella, L. monocytogenes* and *E. coli* O157:H7) were associated with 86.5% of outbreaks followed by parasites (11.6%) and viruses (1.9%). Outbreaks were associated with a variety of domestic and imported vegetables; however, the majority of the outbreaks were linked with sprouts and leafy greens. Using standard vegetable-processing practices, it is not possible to completely inactivate human pathogens that may contaminate a commodity. Because vegetables are often consumed raw and minimally processed, the potential human health risks increase (Beuchat, 2006; Olaimat and Holley, 2012). Clear strategies must be developed to identify sources of pathogens, and steps must be done to prevent contamination of vegetables throughout the production chain (Doyle and Erickson, 2012). Sources of vegetable contamination will be partitioned to pre-harvest (focused on soil amendments, irrigation water, climate change and geographical location) and post-harvest (harvesting, handling and processing). Current knowledge to future strategies to improve the microbial safety of fresh and fresh-cut vegetables will be discussed.

### Pre-harvest

Potential sources of pathogen contamination in pre-harvest include soil, wildlife faeces, soil amendments, agricultural water, reconstituted fungicide and insecticides, dust, wild or domestic animals, field workers, and harvesting equipment (Beuchat, 2006; Matthews, 2013; 2014). Pathogenic bacteria mainly associated with produce outbreaks such as *E. coli* O157:H7, *Salmonella* and *L*. *monocytogenes* are known to be shed into the environment through animal hosts (Ivanek *et al*., 2006; Franz and van Bruggen, 2008). Research has demonstrated the long-term survival of enteric pathogens in agricultural waters, soil amendments and soil (Franz *et al*., 2005; Scott *et al*., 2006; Franz and van Bruggen, 2008). Studies are now being conducted under field conditions, rather than in a greenhouse, potentially providing a better understanding of how enteric pathogens behave in a production environment (Matthews, 2013).

### Manure and soil amendments

Research demonstrates that animal manure used as soil amendments may contain viruses, parasites or bacteria that represent a human health concern. The faeces of wild life must also be considered. Application of manure as a soil amendment would be broadcast as a solid, semi-solid or liquid throughout the field, whereas faeces from wildlife would be introduced at distinct locations. In a large outbreak of *E. coli* O157:H7 associated with spinach in the United States, environmental investigation revealed through molecular typing that isolates from faeces of feral swine matched the outbreak strain (Jay *et al*., 2007). Methods to prevent wildlife intrusion of a field can be costly and of limited effect. Fencing off a production field is costly, often not practical, and if not done correctly, wild pigs and other animals can burrow under the fence. Field workers can monitor field intrusion through detection of animal tracks and disruption of crops. Practical cost appropriate methods are needed to mitigate this source of crop contamination.

Pellet forms of manure (e.g. chicken manure pellets) are widely used as a soil amendment. Pelletizing the manure reduces offensive odour and facilitates storage and transport (Chen and Jiang, 2014). Not surprisingly, chicken litter can harbour a diverse range of pathogens (*Campylobacter*, *Clostridium*, *Salmonella* and *Staphylococcus*), and if not properly treated prior to field application, can result in crop contamination (Ngodigha and Owen, 2009; Silva *et al*., 2009; Chinivasagam *et al*., 2010). During manufacture of manure pellets, a thermal process is performed; however, few studies have been conducted to validate whether the process inactivates microbes of human health concern. Process validation studies would facilitate development of standardized practices that would increase the microbial safety of pellet forms of manure.

The use of fish emulsion applied to agricultural production fields as a means to increase soil nitrogen has increased in popularity. There are a number of methods to prepare the fish emulsion some of which include a thermal process. Similar to the concern with pelletized manure, research to validate the inactivation of human pathogens (bacteria, viruses) by the process is essential.

### Irrigation water

Irrigation is considered as one of the most important transmission modes of enteric human pathogens to vegetable crops (Park *et al*., 2012). Monitoring of presence or absence of human pathogens or testing of indicator microorganisms is done to determine the satisfactoriness of agricultural waters applied to crops (Gerba, 2009). In developing countries, untreated sewage water is often used for irrigation of vegetables. The sampling of ready-to-eat (RTE) salads composed of raw vegetables in Pachuca-City, Hidalgo, Mexico, where most locally consumed vegetables are irrigated with untreated sewage water, found faecal coliforms (99%), *E. coli* (85%) and diarrheagenic *E. coli* (7%) associated with the 130 salad samples tested (Castro-Rosas *et al*., 2012). The World Health Organization recommends that the level of faecal coliforms in wastewater be used for produce irrigation not exceeding 1000 colony-forming units (cfu) or most probable number (MPN) MPN 100 ml^–1^ (World Health Organization/Food and Agriculture Organization, 2006). The results underscore that the use of untreated wastewater in crop production results in poor microbiological quality of RTE salads, which can present a human health risk.

River water used for irrigation can also harbour microorganism that cause human illness. Counts of faecal coliforms and *E. coli* in river water used in vegetable irrigation in South Africa were up to 1.6 × 10^6^ cfu 100 ml^–1^ and 3.5 × 10^5^ cfu 100 ml^–1^ respectively (Gemmell and Schmidt, 2012). A study of pond and tidal creek water in Australia found water samples positive for genes suggesting the presence of *E. coli* O157, *Campylobcter jejuni* and *Salmonella* (Ahmed *et al*., 2009). *Salmonella* were isolated from 57 of 72 (79.2%) surface water samples collected in Georgia, USA (Haley *et al*., 2009). In Canada, *E. coli* O157:H7 and *Salmonella* were detected in samples of river and canal water (Gannon *et al*., 2004). The strain of *Salmonella* Newport linked to an outbreak of tomato-associated salmonellosis was isolated from pond water used for irrigating tomatoes linked to the outbreak (Greene *et al*., 2008). Regardless of whether vegetable production is occurring in a developed or developing country, water used for irrigation must be of suitable microbiological quality. In-line systems to treat irrigation water immediately prior to field application are available. In-line or similar methods to treat irrigation water that are cost-effective and suitable for use in remote locations are needed.

The risk of microbiological contamination of the edible portion of a crop is directly impacted by the method of irrigation: surface furrow, subsurface drip or overhead sprinkler. Research confirms that sprinkle irrigation compared with furrow and subsurface drip increased the risk of crop contamination (Fonseca *et al*., 2011 reviewed by Matthews, 2014). Greenhouse and laboratory studies showed that *E. coli* O157:H7 persisted for 20 days on lettuce leaves that were spray irrigated and that repeated irrigation with contaminated water increased level of the pathogen on the edible portion of the lettuce (Solomon *et al*., 2002; 2003). Van der Linden and colleagues (2013) demonstrated that overhead sprinkle irrigation was associated with increased enteric pathogen contamination of butterhead lettuce. Repeated spray irrigation with a low dose (approximately 3.5 log CFU ml^−1^) of *E. coli* O157:H7 resulted in internalization of the pathogen in spinach and parsley leaves, but not in lettuce leaves (Erickson *et al*., 2014). The pathogen persisted for 2 days post-exposure, suggesting that irrigation immediately prior to harvest presents an increased microbiological risk. The establishment of appropriate guidelines for pre-harvest application of irrigation water is difficult, considering the spectrum of results from research studies addressing the issue (Matthews, 2013; 2014).

### Climate change and geographical location

The issue of climate change associated with food safety has received increased attention (Miraglia *et al*., 2009; Lake *et al*., 2012). Fresh vegetables are cultivated and harvested under diverse climatic and geographical conditions. When discussing microbial safety of vegetables environmental conditions influenced through climatic change or geographic region should be considered because such changes can influence genetic, phenotypic and physiological characteristics of microbes that typically contaminate vegetables in the field (Liu *et al*., 2013). Changes in annual temperature and precipitation patterns have been observed worldwide (Meehl *et al*., 2007). The global average temperature has gradually increased since the earliest observations (Camuffo and Bertolin, 2012). Based on climate-modelling studies, the amount of precipitation is projected to increase in some areas (e.g. tropical and high latitude regions) and decrease in other areas (e.g. Central and Southern Europe, Central North America, Central America and Mexico and Southern Africa), which implies that the characteristics of climate change may vary geographically (Intergovenmental Panel on Climate Change (IPCC), 2012).

The transfer, prevalence and survival of enteric pathogens in soil amendments, soil, agricultural water and on crops may individually or collectively be influenced by climate change or geographic location (Liu *et al*., 2013). The survival of *E. coli* O157 and *Salmonella* in manure and soil decreased as ambient temperature increased (Mukherjee *et al*., 2006; Semenov *et al*., 2007; Danyluk *et al*., 2008). Persistence of *E. coli* O157:H7 and *S.* Typhimurium in manure and manure-amended soil under tropical climatic conditions was shown to be shorter compared with survival time previously observed in temperate regions (Ongeng *et al*., 2011). Survival of *E. coli* O157:H7 in leafy green producing soils in California differed from those in Arizona, which could be attributed to different soil characteristics (salinity, assimilable organic carbon and total nitrogen) within the geographic locations (Ma *et al*., 2012). Increased intensity of precipitation may enhance survival of pathogens in soil and manure by increasing moisture content (Warriner *et al*., 2009). Greater intensity of rain events may lead to vegetable contamination with pathogens by splashing manure and soil particles onto edible portions of the crop or by broadcasting pathogens throughout the field during flooding events (Cevallos-Cevallos *et al*., 2012). Internalization of *S.* Typhimurium by lettuce was shown to be facilitated under experimental conditions of extreme weather such as storms (heavy rainfall) and drought (Ge *et al*., 2012). Seasonality has been demonstrated to affect microbial populations and survival of pathogens associated with vegetables (Ailes *et al*., 2008; Oliveira *et al*., 2012) and abundance of pathogens or faecal coliforms in irrigation water sources (Haley *et al*., 2009; Abakpa *et al*., 2013). Collectively, these studies indicate that a wide range of climatic and geographic variables must be considered when developing strategies to enhance the microbial safety of crops in the field. A ‘one-size fits all’ approach will not work.

### Viruses and the pre-harvest environment

The fate of viruses in water, manure, soil and pre-harvest contamination of crops is largely unknown. Research addressing the issue is grossly lacking as highlighted in a comprehensive review on sources of viruses and viral contamination of crops (Wei and Kniel, 2010). Viruses being shed from human and animal faeces may contaminate soils, water and manure. Viruses associated with leakage from septic tanks, run-off from animal lagoons, or that survive manure treatment may contaminate vegetable production fields and consequently crops. Manure management systems provide liquid, slurry, semi-solid or solid manure that can be applied to agricultural land. Unfortunately, these systems do not completely eliminate viruses (Wei and Kniel, 2010). Because the infectious dose of some viruses is 10–100 particles, pre-harvest contamination of vegetable crops with viruses presents a significant human health concern.

## Harvesting/post-harvest practices and contamination

### Harvesting practices and handling prior to processing

Cross-contamination of vegetables can occur during harvest through contact with harvesting equipments, knives, workers' hands or gloves and containers such as bins, boxes and buckets (Matthews, 2013). Field trimming and coring of lettuce during harvest has been widely adopted because it reduces costs of shipping and waste disposal. However, the practice of core removal can bring tissue damage and additional human handling, which increases microbial infiltration through the cut edges, microbial growth and opportunities of lettuce contamination (Food and Agriculture Organization/World Health Organization, 2008). Taormina and colleagues (2009) reported that coring knives can transfer *E. coli* O157:H7 to lettuce heads after contact with pathogen-contaminated soil. Coring knives can also be contaminated with enteric pathogens through contact with workers' gloves. Machine harvesting has been increasingly used, but it can increase the chance for surface contact exposure (Fallon *et al*., 2011). Lawn mower-type machines commercially used in harvesting baby spinach can introduce microbial hazards, including foodborne pathogens, to the raw product by contact with contaminated soil or manure (Buchholz *et al*., 2012). Accordingly, harvesting tools and machines should be kept clean through periodic washing and sanitizing to prevent the risk of cross-contamination.

Pathogens that may be present on hands can be transferred to vegetables or food contact surfaces (Todd *et al*., 2008; 2009). Bidirectional transfer of *S.* Typhimurium between bare/gloved hands and green bell pepper was demonstrated (Jimenez *et al*., 2007). The study showed that an effective log reduction of *S.* Typhimurium on hands could be achieved using a combination of hand washing and application of an alcohol-based hand gel. To minimize the risk of cross-contamination by hands or gloves, appropriate hand-washing and bathroom facilities should be provided at work sites, without creating a potential hazard for contamination of nearby crops.

Harvested vegetables can be contaminated as the result of improper handling during storage and transportation. Baby greens are harvested into bins, and before transport to the processing facility, the bins are often directly placed onto soil and stacked on the top of other bins, which can permit transfer of contaminants during stacking (Matthews, 2014). A change in practices is required, emphasizing that bins should not be placed on the ground nor stacked after filled.

Rapid cooling of vegetables after harvest is a significant factor for quality maintenance. Cooling vegetables to refrigeration temperature (4°C or less) will decrease not only the rate of respiration and the ripening process, but also the growth of enteric pathogens (Buchholz *et al*., 2010; Matthews, 2014). Several methods of pre-cooling are available and are used depending on commodity. Methods include room cooling (e.g. tomatoes), forced air cooling (e.g. tomatoes and peppers), hydrocooling (e.g. leafy greens, carrots, asparagus) and vacuum cooling (e.g. leafy greens, pepper) (US Department of Agriculture/Agricultural Research Service, 2004). Hydrocooling and vacuum cooling are commonly used in rapid cooling for leafy greens by showering the product with cold water and surface water evaporation respectively (Buchholz *et al*., 2010). Those cooling processes may lead to commodity cross-contamination or internalization of pathogens. Li and colleagues (2008) reported that the vacuum cooling facilitates infiltration of *E. coli* O157:H7 into lettuce. Refrigerated storage limits prevent the growth of some foodborne pathogens; however, most are capable of survival during cold storage (Koseki and Isobe, 2005; Hsu *et al*., 2006; Khalil and Frank, 2010).

### Processing (cutting, washing/sanitizing and packaging/storing)

Processing operations provide many opportunities for cross-contamination because cutting, washing and sanitizing, packaging, and storing are involved. Cutting of vegetables releases exudates-containing nutrients that can aid in the growth of enteric pathogens (Lynch *et al*., 2009; Matthews, 2013). Tissue damage associated with cutting and shredding of lettuce resulted in an increase in the population of *E. coli* O157:H7, likely related to release of latex from the cut surface (Brandl, 2008). Investigators have evaluated the persistence or transfer of viruses in food processing and preparation environments (Fallahi and Mattison, 2011; Stals *et al*., 2013; Wang *et al*., 2013). Norovirus and hepatitis A virus were transferred from contaminated produce (e.g. tomato, cucumber and carrot) to utensils (knives and graters) after cutting and grating (Wang *et al*., 2013).

Washing of vegetables removes soil and other debris and has the added benefit of reducing microbial load (Gil *et al*., 2009). Wash water of unsatisfactory microbial quality or not treated with a sanitizer can serve as a vehicle for dispersion of microorganisms (Holvoet *et al*., 2012). Washing was the primary vehicle for the homogenous spread of *Salmonella* Enteritidis to fresh-cut vegetables during processing (Pérez-Rodríguez *et al*., 2014). Chlorine-based sanitizers are widely used in the fresh produce industry because of their relatively low cost. The potential impact of chlorine-based sanitizers on human health and the release of hazardous by-products into the environment have hastened the research for alternative, safe and cost-effective sanitizers. Optimization of equipment design and processing practices for washing and sanitizing are essential for increasing microbial reduction and ensuring microbial safety of fresh vegetables (Matthews, 2013).

Unfavourable conditions during packaging and storage contribute to the growth and survival of spoilage and pathogenic microorganisms on vegetables (Capozzi *et al*., 2009; Caleb *et al*., 2013). The growth of pathogens (*E. coli* O157:H7, *Salmonella* and *L. monocytogenes*) increased on shredded lettuce stored at abuse temperature (25°C) under modified atmosphere packaging conditions (Oliveira *et al*., 2010). The populations of *Salmonella* and *L. monocytogenes* increased on RTE vegetables (e.g. arugula, collard greens, escarole and spinach) held at abuse temperature (15°C) (Sant'Ana *et al*., 2012). Change in packaging conditions or storage temperature has been reported to influence expression of virulence factors of pathogens (Carey *et al*., 2009; Sharma *et al*., 2011). An increase in expression of *E. coli* O157:H7 virulence genes, *eae*, *stx2* and *iha* were observed in cells that were associated with lettuce held under abuse storage temperature and near-ambient air atmosphere conditions (Sharma *et al*., 2011). Collectively, contamination with pathogenic microbes and increased spoilage microbial load on a product can occur during post-harvest processing of vegetables if best practices are not employed.

### Outbreaks – The source of contamination

Identifying the point or source of contamination for a given outbreak is difficult at best, and for most outbreaks, the specific vehicle or source of contamination is not determined (Table 1). The complexity of a given product's journey from field to consumer makes identifying the point of contamination difficult and is exacerbated when the products come from foreign sources. Several outbreaks are presented to underscore the importance of the implementation of effective production and processing practices to provide healthy and microbiologically safe vegetables to the consumer.

**Table 1:** Selected foodborne outbreaks linked to vegetables

Year	Implicated vehicle	Aetiology	Number of cases (hospitalization %)	Finding during environmental investigation
2014	Raw clover sprouts	*E. coli* O121	18 (44)	Unsanitary condition
2013	Ready-to-eat-salad	*E. coli* O157:H7	33 (32)	There were 5 of 10 samples from the surrounding areas of the harvest field that were positive for *E. coli* O157:H7, but do not match the outbreak strain
	Fresh produce (salad mix and cilantro)	*Cyclospora*	631 (8)	*Cyclospora* were not detected from environmental samples
	Cucumbers	*S.* Saintpaul	84 (28)	Investigation is still going on
2012	Spinach and spring mix	*E. coli* O157:H7	33 (46)	Source of contamination has not been identified yet
	Raw clover sprouts	*E. coli* O26	29 (33)	Potentially contaminated seeds
2011	Romaine lettuce	*E. coli* O157:H7	60 (67)	Contamination source was not identified
	Alfalfa and spicy sprouts	*S.* Enteritidis	25 (30)	Samples did not yield *Salmonella*, but observed unsanitary conditions
2010	Alfalfa sprouts	*Salmonella* I 4,(5),12:i:-	140 (24)	Unsanitary condition, one environmental (water run-off) sample yielded outbreak strain
	Alfalfa sprouts	*S.* Newport	44 (19)	Contaminated seeds
	Shredded romaine lettuce	*E. coli* O145	33 (40)	No deficiencies in the producer's good agricultural practices found – O145 outbreak strain was not detected
2009	Alfalfa sprouts	*S.* Saintpaul	235 (3)	Contaminated seeds
2008	Raw produce (jalapeño peppers, serrano peppers, tomatoes)	*S.* Saintpaul	1442 (20)	Irrigation water
2006	Tomatoes	*S.* Typhimurium	190 (22)	Contamination source was not determined
	Iceberg lettuce	*E. coli O157:H7*	71 (75)	No identifiable risk factors were observed
	Fresh bagged spinach	*E. coli* O157:H7	205 (57)	Samples from faeces of cattle and wild pigs and water at ranch were matched to the outbreak strain

### Sprouts

In the United States, during the period from 2006 to 2014, 16 of 68 multistate foodborne outbreaks were linked to vegetables (CDC, 2014a). Seed sprouts were the implicated vehicle in nearly 38% (6 of 16) of those outbreaks. Contaminated seeds or unsanitary conditions of the production facility are often the cause of sprout contamination. Investigation on the 2010 *Salmonella enterica* serotype 4,5,12:i: − alfalfa sprout outbreak identified an environmental sample (water run-off) that was indistinguishable from the outbreak strain (CDC, 2011). During inspection of the production facility, several unsanitary conditions were observed (Food and Drug Administration, 2011). Hazardous conditions included but were not limited to: run-off water (sample was positive for the outbreak strain) from the compost area flowing into a drain near the entrance of the production greenhouse; an employee entered the compost site to dump production waste and returned to the production area without change of clothing, boots nor passage through a footbath with sanitizer; a cart used for transport of sprout trays was moved through the compost area by employees and not cleaned nor disinfected prior to entry back into the sprout-growing area; and no documentation to demonstrate that the seed-sanitizing method used was equivalent to the use of 20 000-ppm calcium hypochlorite recommended by the USFDA. Implementation of measures designed to limit or prevent contamination and cross-contamination of sprouts likely would have prevented the outbreak.

The large 2011 *E. coli* O104:H4 outbreak that was centred in Germany resulted in more than 4000 illnesses, over 850 cases of haemolytic uremic syndrome and 54 deaths (Frank *et al*., 2011). The outbreak was linked to the consumption of fenugreek sprouts; the epidemiological investigation suggested that the seeds were contaminated with a pathogen that grew during sprout production. Methods designed to improve the microbial quality of seeds are required. Present recommendations indicating the soaking of seeds in a high concentration of sanitizer are not completely in effect.

A recent multistate outbreak of Shiga toxin-producing *E. coli* O121 infections was linked to consumption of raw clover sprouts (CDC, 2014b). Even though no outbreak strain was isolated in the facility, FDA inspectors found unsanitary conditions in the facility, including: rusty and corroded water system; condensate dripping directly into the vats containing growing sprouts; inappropriate use of pitchfork and tennis rackets and cracked, damaged and chipped food contact surface (Food and Drug Administration, 2014).

In 2010, a *Salmonella* Newport outbreak was linked to consumption of alfalfa sprouts (CalFERT, 2011). The sprout producer implemented practices based on USFDA guidelines, such as storage of seeds under dry conditions and sanitizing seeds with calcium hypochlorite. Sanitation standard operating procedures were implemented correctly. Footbaths containing quaternary ammonia solution were placed at the entrance of each processing room. All records were daily documented and maintained. No violation nor unsanitary condition was observed. Moreover, environmental swabs, sprout products and germinating seed samples were negative for *Salmonella*. The outbreak *Salmonella* Newport strain was isolated from some seed samples. Contaminated seed was considered as a source of this outbreak.

### Tomatoes and leafy greens

Tomatoes and leafy greens have been linked to a number of well-publicized foodborne outbreaks in the United States and globally. A multistate outbreak of *S.* Saintpaul resulted in more than 1400 infected people and serves to underscore how complicated it can be to delineate the commodity involved and the source of contamination of an outbreak (CDC, 2008). Tomatoes were initially suspected as the vehicle of the outbreak; however, the epidemiological investigation by Centers for Disease Control and Prevention (CDC) and FDA ultimately revealed that the main cause were jalapeño and serrano peppers contaminated with *S.* Saintpaul (Klontz *et al*., 2010). The failure to initially identify peppers as the outbreak vehicle of *S.* Saintpaul resulted in huge economic loss to tomato growers. Behravesh and colleagues (2011) found that 86% of cases were associated with eating salsa, guacamole and a raw jalapeño pepper garnish at Mexican-style restaurants. Further investigation revealed that the jalapeño peppers were contaminated prior to arrival at the restaurant (Mody *et al*., 2011). Traceback investigations included farms, packing operations and products imported to Mexico, Florida and Texas. Jalapeño peppers received by an importer in Texas from a packing facility in Mexico were tested positive for the outbreak strain (Behravesh *et al*., 2011). The outbreak strain was isolated from irrigation water, and serrano peppers on one of the farms where jalapeño peppers involved in the outbreak were grown. Mody and colleagues (2011) concluded that agricultural water was one possible source of contamination and highlighted that water should be protected from animal and waste run-off.

Water as a vehicle for contamination of crops in the field was highlighted in the investigation of the 2006 *E. coli* O157:H7 outbreak associated with pre-bagged spinach. Change in the level of groundwater and ground-surface water was noted as contributing factors in the contamination of crops linked to the outbreak (CDC, 2007). Potential access of cattle and wild pigs to a nearby river was proposed as an additional risk factor in water contamination with *E. coli* O157:H7. Monitoring the satisfactoriness of agricultural waters prior to field application is essential.

In 2013, a multistate outbreak of *E. coli* O157:H7 was linked to RTE salad (CDC, 2013). The traceback investigation linked romaine lettuce from a farm in California as the vehicle for the outbreak. The outbreak resulted in the recall of approximately 181 620 pounds of RTE salad mix (Food and Drug Administration, 2013a). The FDA and The California Department of Public Health (CDPH) conducted an environmental investigation of the farm and found that 5 of 10 soil and water samples from the harvest field and in near proximity of the farm were positive for *E. coli* O157:H7, but not the outbreak strain. Investigators also indicated that two cattle operations in close proximity to the harvest fields may serve as source of contamination and advised the implementation of measures for the prevention of cross-contamination from those operations.

Since the mid-1990s, foodborne cyclosporiasis cases in North America have steadily increased (Kozak *et al*., 2013). In the United States, cyclosporiasis was linked to the consumption of *Cyclospora*-contaminated fresh raspberries, mesclun lettuce and basil (Ho *et al*., 2002). In 2013, a large *Cyclospora* outbreak was associated with salad mix and cilantro (Food and Drug Administration, 2013b). A total of 631 cases were identified from 25 states, and 8% of infected people were hospitalized. The packing facility's employees and restrooms were initially considered as potential sources of *C. cayetanensis*, but were later ruled out. The USFDA conducted an environmental assessment that included harvest crews, ranch workers, onsite sanitary facilities, irrigation water and field samples from five ranches. One potential cross-contamination point was identified as associated with the portable sanitary facilities. The hand-washing faucet, a hose–spigot style handle, should be touched before and after washing hands by field workers. However, all of the approximately 835 product, water and environmental samples tested were negative for *Cyclospora.*

## Conclusion

There is a need for continued research to support development of ‘best practices’ for production and processing of fresh and fresh-cut vegetables to improve the microbial quality and safety of those commodities. Soil amendments can serve as a source of pathogens that cause human illness. Research has focused on solid, slurry and liquid manure types and the effects of manure collection and management systems in pathogen survival. Research is lacking on the microbial safety of fish emulsion and pellet forms of manure (e.g. chicken manure pellets) used as soil amendments. During manufacture, a thermal process may be used, but studies to validate whether pathogens of human importance are inactivated are lacking.

Epidemiological investigations of foodborne illness outbreaks linked to consumption of fresh vegetables often implicate agricultural water as a source of contamination. Comparison of the three methods of irrigation confirmed (overhead sprinkler, subsurface drip or surface furrow) that sprinkle irrigation increased the risk for crop contamination. The literature provides no clear results in, for example, pathogen survival and the appropriate period between a final irrigation event and crop harvest. Improvement in testing and monitoring methods of agricultural waters and development of effective cost-appropriate systems to treat irrigation water prior to crop contact are needed.

A review of the literature indicates that there is a need for greater research on viruses and seed sprout production. Monitoring of soil amendments and agriculture water seldom includes testing for viruses or validation that the treatment processes inactivate viruses. Globally, seed sprouts have been linked to devastating outbreaks of foodborne illnesses. In many outbreaks, the source of contamination was traced to the seeds. Novel seed production and processing methods appropriate for use by conventional and organic producers are needed to improve the microbial safety of sprouts.
